# Genome Engineering with TALE and CRISPR Systems in Neuroscience

**DOI:** 10.3389/fgene.2016.00047

**Published:** 2016-04-06

**Authors:** Han B. Lee, Brynn N. Sundberg, Ashley N. Sigafoos, Karl J. Clark

**Affiliations:** ^1^Neurobiology of Disease Graduate Program, Mayo Graduate SchoolRochester, MN, USA; ^2^Department of Biochemistry and Molecular Biology, Mayo ClinicRochester, MN, USA

**Keywords:** ZFN, TALE, TALEN, CRISPR, Cas9, genome engineering, neuroscience

## Abstract

Recent advancement in genome engineering technology is changing the landscape of biological research and providing neuroscientists with an opportunity to develop new methodologies to ask critical research questions. This advancement is highlighted by the increased use of programmable DNA-binding agents (PDBAs) such as transcription activator-like effector (TALE) and RNA-guided clustered regularly interspaced short palindromic repeats (CRISPR)/CRISPR associated (Cas) systems. These PDBAs fused or co-expressed with various effector domains allow precise modification of genomic sequences and gene expression levels. These technologies mirror and extend beyond classic gene targeting methods contributing to the development of novel tools for basic and clinical neuroscience. In this Review, we discuss the recent development in genome engineering and potential applications of this technology in the field of neuroscience.

## Introduction

The relationship between genotype and phenotype is often not direct and one-to-one (Hirschhorn and Daly, 2005; Manolio et al., 2009). In clinical settings, analyzing patients' whole genome sequencing data and triaging the significance of a variant remain a challenging task (MacArthur et al., 2014; Shendure, 2014; Richards et al., 2015). This is especially the case for neural or behavioral phenotypes. Many neuropsychiatric disorders are multi-genic or multi-factorial involving complex gene-gene or gene-environment interactions (Franke et al., 2009; Sullivan et al., 2012; Dunn et al., 2015). Disease manifestations of neuronal diseases, whether neuropsychiatric or neurodegenerative, are often circuit- or neuronal population-specific making circuit-level understanding indispensable in neuroscience (Shin and Liberzon, 2009; Russo and Nestler, 2013; Shepherd, 2013). Neuronal subtypes are diverse, and each type may show distinct structural, functional, electrophysiological, and connectional properties. Different methods estimate the number of neuronal subtypes in the brain from ~100 to 1000 (Stevens, 1998; Masland, 2001, 2004; Nelson et al., 2006). Different epigenetic landscapes endow a distinct identity to each neuronal subtype that arises from a common genome within each organism. This epigenetic encoding could be the substrate in which the gene-environment interaction is consolidated (Vialou et al., 2013). Interrogating such complexity in the nervous system requires sophisticated methodologies that can manipulate each component while minimizing the perturbation of the whole system. Recently developed genome engineering technologies allow genomic perturbations with surgical precision, and these technologies are well-suited for advancing neuroscience.

There are several advantages to genome engineering technologies. First, programmable DNA-binding agents (PDBAs) can be targeted to any locus in the genome. Particularly, transcription activator-like effector (TALE) and clustered regularly interspaced short palindromic repeats (CRISPR)/CRISPR associated (Cas) systems can be targeted at nucleotide resolution. Second, a variety of effectors can be fused to the PDBA as a module. PDBAs fused to endonucleases make breaks in the DNA that can introduce random or targeted DNA changes when co-delivered with exogenous DNA sequences. With transcription activators and repressors, or epigenetic modifiers, gene expression can be up- or down-regulated. Using fluorescent proteins as an effector, specific genomic loci can be visualized in live cells or animals allowing for structural and organizational investigations of chromatin. Third, PDBAs can be used for therapeutic purposes with applications ranging from correcting mutations in monogenic disorders to modulating gene expression. In this Review, we discuss recent developments in genome engineering and their potential applications in the field of neuroscience. We summarize the use of PDBAs as (1) genome editing tools and (2) gene expression modulators, and discuss (3) other considerations in applying these systems in neuroscience.

## Genome editing with customizable endonucleases

The field of genome engineering has developed over the past several decades. Classic gene targeting that has established mutant mouse models was the first to show that biological functions of the genes can be perturbed and studied *in vivo* (Evans and Kaufman, 1981; Smithies et al., 1985; Thomas and Capecchi, 1986). Around the same time, investigations into nonhomologous end joining (NHEJ) and homologous recombination (HR) were defining and uncovering DNA repair pathways (Roth and Wilson, 1985; Lehman et al., 1993; Phillips and Morgan, 1994; Hagmann et al., 1996). The biology of gene targeting and DNA repair mechanisms were gradually elucidated, uncovering critical variables for genome engineering such as linear donor DNA is more recombinogenic than circular ones (Orr-Weaver et al., 1981; Folger et al., 1982; Rong and Golic, 2001) or a DNA double-strand break (DSB) in the genome increases the rate of HR (Puchta et al., 1993; Rouet et al., 1994; Choulika et al., 1995; Smih et al., 1995). The structural studies and engineering of zinc finger protein led to the development of first programmable DNA binding agents (Pavletich and Pabo, 1991; Desjarlais and Berg, 1992; Choo and Klug, 1994; Wu et al., 1995; Kim et al., 1996; Smith et al., 2000).

### Zinc finger nucleases (ZFNs)

The zinc finger nuclease (ZFN) was introduced in 1996 to address the need for more specific restriction endonucleases (Kim et al., 1996). Repeated zinc finger DNA-binding domains were fused to a FokI endonuclease domain to allow for programmable DNA sequence binding specificity as each finger motif moves along the major groove of the DNA double helix and stabilizes upon binding three matching base pairs (Kim et al., 1996).

In the early 1990s it was shown that HR increased by two- to four- orders of magnitude in plant and mammalian cells when a double-strand break (DSB) was introduced to the genome by the rare-cutting endonuclease, I-*Sce*I (Puchta et al., 1993; Rouet et al., 1994; Choulika et al., 1995; Smih et al., 1995). Exploiting this, a collaboration between Chandrasegaran and Carroll labs showed that HR could be significantly increased when ZFNs made DSBs in the genome (Bibikova et al., 2001).

Subsequent refinement of ZFNs provided a template for innovation of next generation genome engineering tools–TALENs and CRISPR/Cas9. For example, modified FokI catalytic domains, which increased specificity or efficiency, were first developed for ZFN applications and later adapted for TALENs. Obligate heterodimeric versions of FokI significantly decreased off-target effects and genome toxicity caused by unintended homodimerization (Miller et al., 2007; Szczepek et al., 2007). Guo et al. used directed evolution to select for increased FokI activity, improving mutagenic efficiency by three- to six-fold (Guo et al., 2010). A zinc finger nickase system had been shown to favor homology-directed repair (HDR) without activating NHEJ repair, minimizing opportunities for errors and off-target effects (Kim et al., 2012; Ramirez et al., 2012). A nickase commonly refers to an endonuclease that generates a single-strand DNA break, nick.

ZFNs have been widely used to create animal models and are currently the only genome editing tool being utilized in clinical settings. Studies addressing diseases such as Huntington's disease (Garriga-Canut et al., 2012) and Hurler and Hunter syndromes (Sharma et al., 2015) are in pre-clinical development, and HIV treatments have reached early clinical trials (Perez et al., 2008; Wilen et al., 2011; Tebas et al., 2014; for a review, see Jo et al., 2015). ZFN research is now focused on improving delivery methods. Despite the pioneering role of ZFNs, difficulty of designing and optimizing the ZFN reagents (Greisman and Pabo, 1997) led to rapid adaptation of first TALEN and then CRISPR/Cas9 systems.

### Transcription activator-like effector nucleases (TALENs)

**Figure 1:**
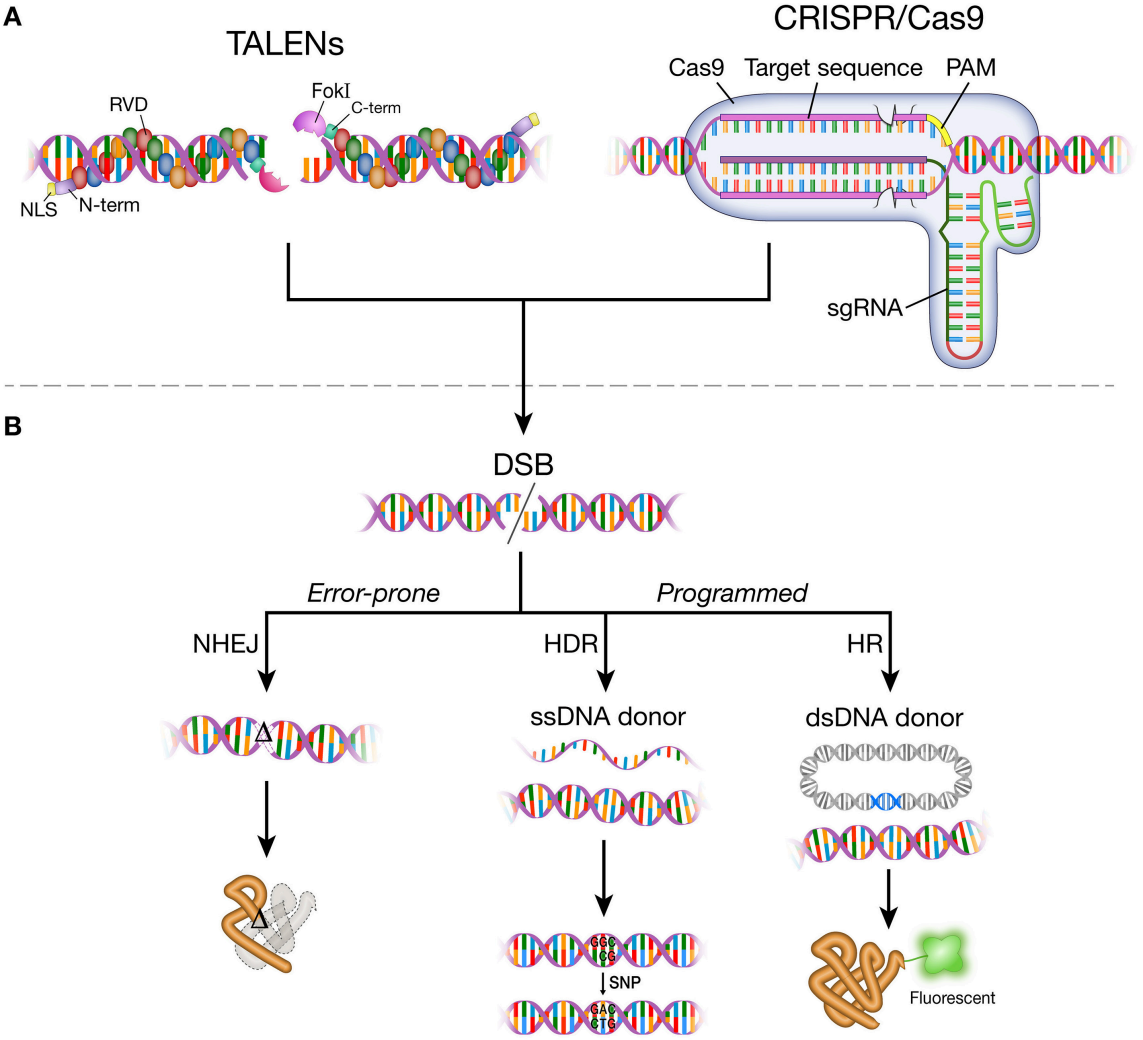
**DNA-binding agents, DNA double-strand break (DSB) and repair pathways**. **(A)** A pair of TALEN monomers bound to double-strand DNA. A TALEN monomer comprises N-terminal domain, nuclear localization signal, modular repeats that contain two highly variable amino acid residues (RVDs), C-terminal domain, and FokI endonuclease. TALENs bind to target DNA in the major groove. A pair of TALEN monomers targeted closely enable FokI dimerization to make DNA double-strand break. Cas9 protein and sgRNA form a complex. The sgRNA-Cas9 complex bind to target DNA through Watson-Crick base-pairing between the target recognition region of the sgRNA and the target DNA sequence (protospacer). The target recognition region (pink rectangle) is the RNA sequence in the sgRNA that matches the target DNA sequence (20 nt). The PAM (protospacer adjacent motif) sequence varies depending on the type of Cas protein. *Streptococcus pyogenes* Cas9 protein recognizes 5′-NGG-3′ sequence. **(B)** Targetable TALEN and CRISPR/Cas9 systems generate DNA double-strand break (DSB) at the target locus in the genome. DSBs are repaired by non-homologous end joining (NHEJ), homology-directed repair (HDR), or homologous recombination (HR). Error-prone NHEJ results in non-functional proteins or loss of protein through frameshift mutations introduced to open reading frame. HDR and HR mediate faithful repair of DNA break by incorporating exogenously provided DNA templates in either single strand DNA oligos (ssDNA donor) or plasmids (dsDNA donor). HDR and HR can be used to change small sequences or tag the target gene with a reporter (e.g., green fluorescent protein). TALENs, transcription activator-like effector nucleases; NLS, nuclear localization signal; RVD, repeat variable di-residue; FokI, FokI endonuclease; N-term, N-terminal domain; C-term, C-terminal domain; CRISPR/Cas9, clustered regularly interspaced short palindromic repeats/CRISPR associated protein 9; sgRNA, single guide RNA; PAM, protospacer adjacent motif.

The DNA-binding mechanism of TALEs was elucidated in 2009 (Boch et al., 2009; Moscou and Bogdanove, 2009), years after the discovery of TALEs as secreted proteins from the bacterial plant pathogen *Xanthomonas* (Bai et al., 2000; Yang and White, 2004; Gu et al., 2005; Kay et al., 2007; Sugio et al., 2007). Native TALEs are comprised of an N-terminal domain, central repeats of the DNA binding domain, a C-terminal segment that incorporates nuclear localization signals, and a transcriptional activation domain (Bogdanove et al., 2010). The DNA-binding domain consists of 33–35 amino acid repeats with differences at residues 12 and 13 (repeat variable di-residue; RVD). A specific RVD in the DNA-binding domain recognizes a base in the target locus, providing a structural feature to assemble predictable DNA-binding domains. The DNA binding domains of a TALE are fused to the catalytic domain of a type IIS FokI endonuclease to make a targetable TALE nuclease (TALEN; Figure 1A). To induce site-specific mutation, two individual TALEN arms, separated by a 14–20 base pair spacer region, bring FokI monomers in close proximity to dimerize and produce a targeted DSB (Figure 1A).

Several modifications to the native TALE composition and initial TALEN design have improved binding and cleavage specificity and efficiency (Christian et al., 2010; Hockemeyer et al., 2011; Li et al., 2011; Tesson et al., 2011). In an effort to improve TALEN efficiency, improved TALEN scaffolds incorporated optimized nuclear localization sequences, amino-terminal, and carboxy-terminal truncations, and assorted FokI nuclease linkers (Mussolino and Cathomen, 2012). *In vitro* scaffolding modifications have been studied by fusing variable C-terminal truncations to the catalytic domain of FokI to determine which variants enable efficient cleavage, the final modification of which retained only 28 or 63 of the 278 original C-terminal residues (Miller et al., 2011). Different TALEN scaffolds and native TALEs from other bacterial species continue to be tested and optimized to achieve a higher and more consistent activity of targeting genetic modifications. Our lab has used the GoldyTALEN scaffold to improve *in vivo* efficacy with a messenger RNA expression vector backbone (pT3TS). When using the GoldyTALEN scaffold, there was a six-fold increase in somatic gene modification, and germline modification rate increased from 17 to 71% (Bedell et al., 2012). Achieving a higher level of TALEN activity can depend on the length (base pairs) of the spacer between the two individual TALEN binding sites and the specificity of DNA binding (Ma A. C. et al., 2013a).

### Clustered regularly interspaced short palindromic repeats/CRISPR associated (CRISPR/Cas)

First identified in *Streptococcus pyogenes*, the native type II CRISPR/Cas system is a bacterial adaptive immune response and the simplest among the three CRISPR/Cas systems that requires only four Cas proteins. During exposure to foreign DNA (the acquisition phase), the type II CRISPR/Cas system incorporates a short foreign DNA sequence (protospacer), using Cas1 and Cas2 proteins, into the bacterial genome between short palindromic repeats, thus forming a CRISPR array. The expression phase of the immune response occurs when the CRISPR locus is transcribed into a long non-coding pre-CRISPR RNA (pre-crRNA), along with its partial complement, the trans-activating CRISPR RNA (tracrRNA). The tracrRNA forms base pairs with the repeat sequences of pre-crRNA, and host RNAIII cleaves the repeat sequence to produce mature crRNAs. For DNA cleavage to occur (the interference phase), the crRNA:tracrRNA:Cas9 complex scans the invading DNA for the PAM sequence and, upon Watson-Crick base pairing between crRNA and foreign DNA, neutralizes it via a DSB (for a review, see Sorek et al., 2008; Horvath and Barrangou, 2010; Jiang and Doudna, 2015; Wright et al., 2016).

Since the CRISPR sequence was discovered in 1987 (Ishino et al., 1987), the utility of CRISPR/Cas for targeted DNA cleavage was first demonstrated in 2012 *in vitro* and in bacterial cells (Gasiunas et al., 2012; Jinek et al., 2012). Unique from ZFNs and TALENs, which use protein domains to recognize specific DNA sequences, the CRISPR/Cas system uses RNA-based sequence recognition (Figure 1A). The crRNA and tracrRNA in the native system were simplified into a single guide RNA (sgRNA) of approximately 100 nucleotides with a generic tetraloop secondary structure to improve ease-of-use and customizability for engineering (Jinek et al., 2012). Since the application of CRISPR/Cas systems in mammalian cells (Cho et al., 2013; Cong et al., 2013; Jinek et al., 2013; Mali et al., 2013b), the technology has rapidly evolved: multiplexing in the CRISPR/Cas system (Cong et al., 2013), developing a paired Cas9 nickase system (Shen et al., 2014), dimerizing the dCas9 (dead Cas9; catalytically inactive) system fused to FokI endonuclease (Tsai et al., 2014a), adding a DNA-binding domain for improved target specificity (Bolukbasi et al., 2015), or splitting Cas9 into two components for improved packaging and delivery in a viral vector and temporal control (Truong et al., 2015; Wright et al., 2015; Zetsche et al., 2015b). Other efforts focus on reducing targeting limitations posed by the strict PAM recognition sequence. Different variants of Cas proteins have been shown to reduce targeting limitations (Kleinstiver et al., 2015); for example, a Cas9 ortholog, Cpf1, uses a T-rich PAM sequence (Zetsche et al., 2015a).

With these successive innovations, the CRISPR/Cas9 system has become widely adapted for genome engineering. Some of the reasons for its popularity are the constructs to produce sgRNA can be easily prepared with one cloning process, the Cas9 construct can be re-used without any modification, and targeting multiple loci (multiplexing) is easily achieved by introducing several sgRNAs. The simplicity of sgRNA production has made it possible to generate libraries of knockout guide RNAs for either human or mouse genes that are useful for forward genetic screens (Koike-Yusa et al., 2013; Shalem et al., 2014).

### DNA double-strand break repair in neurons

DNA double-strand breaks (DSBs) induced by targetable endonucleases are only the first step in mutagenesis (Figure 1B). The perturbation of the genomic DNA sequence is useful in genome engineering only because cellular DNA damage signals and repair machineries are activated and fix the damaged sequence. It is the repair process that we exploit to isolate desired disease alleles or to revert mutations to wild-type sequences.

Such DNA repair pathways are rapidly mobilized after damage because maintaining genomic integrity is critical for cell survival and function. DNA damage can occur in many ways. Cell metabolism generates oxidative DNA damage, ultraviolet radiation results in single- or double-strand breaks, and cross-linking agents lead to intra- or inter-strand linkages (Lindahl, 1993; Rao, 1993; Brooks, 2002; Caldecott, 2008; McKinnon, 2013; Pan et al., 2014). Among the many forms of DNA damage, double-strand breaks (DSBs) are considered the most detrimental and are repaired by three major pathways: homologous recombination (HR), homology-directed repair (HDR), and non-homologous end joining (NHEJ) (Figure 1B; Lieber, 2010; Chiruvella et al., 2013; Jasin and Rothstein, 2013). The HR pathway allows an error-free repair and requires an extensive sequence homology from which the sequences of the damaged DNA can be faithfully copied. The sequence homology is provided by the sister chromatid, and thus HR is operational during the S and early G2 phases of the cell cycle during which the sister chromatid is available (Potter et al., 1987; Maryon and Carroll, 1991; Benjamin and Little, 1992; Kadyk and Hartwell, 1992). NHEJ is an error-prone process in which the broken DNA ends are directly ligated through the end processing proteins, Ku70:Ku80 [a part of DNA dependent protein kinase (DNA-PK)], catalytic subunit of DNA-PK (DNA-PKsc), and ligase IV (Critchlow and Jackson, 1998). During this end-joining process, small-scale nucleotide insertions and deletions (indels) are commonly involved (Heidenreich et al., 2003). Since NHEJ does not require a homology sequence, it can occur throughout the cell cycle. HDR broadly defines a wide array of homology-dependent pathways of the DNA repair including single-strand annealing (SSA) and alternative NHEJ, also called microhomology-mediated end joining (MMEJ; He et al., 2015). MMEJ utilizes small homologous sequences (1–25 nt) within either end of the broken DNA (Liang et al., 2008; McVey and Lee, 2008). Like NHEJ, most HDR processes can occur throughout the cell cycle. On the basis that both HDR and HR use homologous sequences, HR can be considered part of HDR (Lieber, 2010). Yet, HR possesses processes unfound in HDR or NHEJ, such as extensive processing of the 5′-end of the DNA lesion and strand invasion by the Rad51-bound 3′-end of the lesion (Maryon and Carroll, 1991; Shinohara et al., 1992; Van Dyck et al., 1999; Jasin and Rothstein, 2013). Since DNA repair is a critical process for cell survival, the various repair pathways can be activated by different damage signals. Although there are proteins dedicated to a particular pathway, many of the signaling and repair molecules are redundant rather than exclusive to a single pathway (Takata et al., 1998; Madabhushi et al., 2014).

DNA repair pathways are utilized in two distinct phases in neurons. During the development of an organism, the highly proliferative neural tissues mainly use HR. At this stage, failure to faithfully repair the genome leads to apoptosis (Waser et al., 1979; Sugo et al., 2000; Orii et al., 2006). Since a single error in progenitor cells can lead to severe failures in a large number of cells, either a complete repair or abortive apoptosis is adaptive for the survival of the organism. Once neurons develop, the mature neurons are post-mitotic and their DNA is no longer replicated. In these mature neurons, the sister chromatid with sequence homology is not available and therefore HR is rare. NHEJ is considered to be the major pathway for DNA repair in adult neurons and apoptosis is not promoted (Sonoda et al., 2006; Fortini and Dogliotti, 2010; Iyama and Wilson, 2013). Whereas global repair activities are down-regulated in post-mitotic neurons, transcription-dependent DNA repair pathways are highly active, and transcribed genes are actively repaired regardless of the type of DNA damage (e.g., single strand or double-strand breaks; Nouspikel and Hanawalt, 2000, 2002). Although there is mounting evidence that connects DNA DSBs to neurodegeneration (Hochegger et al., 2006; Wang et al., 2013; Rulten et al., 2014), it is still not clear how these DSBs lead to neurodegeneration in mature neurons (for a review, see McKinnon, 2013; Madabhushi et al., 2014). Also uncertain is whether it is the type or number of errors that contributes to neurodegeneration.

Recently, it was found that DNA DSBs occur as part of normal neuronal physiology, and these DSBs are closely tied to learning, memory, and novel experience-dependent synaptic changes (Suberbielle et al., 2013; Madabhushi et al., 2015). These DSBs have been demonstrated to be required in the promoter regions of early-response genes—responsible for memory formation and synaptic consolidations—in primary neuron culture and hippocampal slices of Swiss Webster mice, and also in an *in vivo* training paradigm for contextual fear conditioning in 6-month-old mice (Madabhushi et al., 2015). In an exploration of novel environment learning paradigm, the DSBs in hippocampal regions in 4-7-month-old mice are repaired within 24 h of the occurrence (Suberbielle et al., 2013). The pathogenic protein in Alzheimer's disease, amyloid-beta, made DSBs persist beyond the 24-h period in 4-7-month-old human amyloid precursor protein (hAPP) transgenic mice (Suberbielle et al., 2013). In addition, DSB repair protein BRCA1 is depleted in a mouse model of Alzheimer's disease, and the depletion is correlated with cognitive decline in mice compared to wild-type mice (Suberbielle et al., 2015). These findings indicate that functional DSB repair is associated with normal neuronal physiology and impaired DSB repair with pathophysiology.

The distinctive DNA repair mechanisms and physiologic utility of DSBs in neurons warrant further investigation on the consequences of using targetable endonucleases in the nervous system. The majority of studies on the functions of PDBAs and the outcomes of PDBA applications were conducted with dividing cell lines such as immortalized cells (Cermak et al., 2011; Christian et al., 2012; Garg et al., 2012; Cho et al., 2013; Cong et al., 2013; Jinek et al., 2013) or stem cells (Hockemeyer et al., 2011; Cong et al., 2013; Ding et al., 2013a,b; Osborn et al., 2013; Mali et al., 2013b). The result of PDBA-induced DSBs is likely to be different in post-mitotic neurons compared with mitotic cells. For example, a faithful sequence correction might be more difficult in nervous tissues since the incorporation of exogenous DNA might be favorably mediated by HDR or NHEJ, both of which are more error-prone repair processes than HR. Or, it could be that neurons might activate the HR pathway if given exogenous DNA templates. These questions need to be empirically determined. Once detrimental mutations occur by DSBs, the nervous tissue would be more susceptible to degeneration since the restoration of the cell population through cell divisions is unlikely. These considerations need to be taken into account when targeting PDBAs in the nervous system as somatic therapeutic agents. Developing an experimental system with which nervous tissue-specific perturbations can be tested will be invaluable to spur therapeutic PDBA development targeting neuronal disorders.

### NHEJ: knock-outs

Since indels in the NHEJ pathway can lead to frameshift mutations producing nonfunctional truncated proteins and loss of protein, NHEJ has been the predominant mode of PDBA-mediated mutagenesis. In earlier applications of PDBA-mediated targeted NHEJ in neuroscience, neuronal genes are targeted in dividing cells to demonstrate that mutations can be introduced at the target loci. Cong et al. targeted *EMX1, Th*, and *PVALB* implicated in neurodevelopment or neurodegeneration using a Cas9 system in HEK293FT cells (Cong et al., 2013; Table 1). PDBA-mediated NHEJ enabled an important advance in rapid modeling of human diseases in animals and cell lines. With PDBAs, virtually any genetic make-up with a well-defined genetic basis (e.g., monogenic) can be produced to model a disease in terms of genomic sequences, if we set aside for another discussion the caveats of biologically replicating the phenotypes, genetic compensation in germline mutants, and embryonic lethality in essential genes. Several investigations have introduced mutations to neuronal loci in zebrafish using ZFNs (Schmid et al., 2013), TALENs (Bedell et al., 2012; Huang et al., 2013), and a Cas9 system (Hwang et al., 2013), in rats using TALENs (Ferguson et al., 2013), and in mice and rats using a Cas9 system (Li et al., 2013; Tables 1, 2). PDBA-mediated NHEJ has made it possible to model neuropsychological and neurodevelopmental disorders in animals that were previously not possible due to lack of germline competent embryonic stem cells (ESCs). Using TALENs, two groups reported successful modification of the *MECP2* locus implicated in Rett syndrome in non-human primate models (Liu H. et al., 2014; Liu Z. et al., 2014). In these two investigations, only one group saw successful birth of injected animals (Liu H. et al., 2014). The fact that the other group could not maintain the surrogate mother's pregnancy of injected zygotes indicates that mosaic F0 animals might not be viable when the mutagenic efficiency of PDBAs is high. Another group targeted *PPARG*—implicated in neurodegeneration—with a Cas9 system in cynomolgus monkeys (*Macaca fascicularis*; Niu et al., 2014). These genetic perturbations have not been fully investigated yet for their phenotypes, nor the degree to which they model human diseases. More follow-up investigations are expected in which the biological relevance of the models to human diseases is reported.

**Table 1:** **Neuronal target loci: knock-out**.

**References**	Locus^*^	Implication^**^	**System**	**Delivery**	**Goal**	**Perturbation**	**Results**
Keatinge et al., 2015	*gba1*	GD, PD	Zebrafish	1-cell embryo injection of TALEN mRNA	Germline transmission	KO; small indels	*gba1^**c*.1276_1298*del**^* mutant line
Yao et al., 2015	*Gpr52*	HD	Mouse	Zygote injection of TALEN mRNA	Germline transmission	KO; small indels	Indel mutants
Mehrabian et al., 2014	*Prnp*	Prion diseases, AD	N2a, C2C12, NMuMG cells	Cas9 and sgRNA plasmid co-transfection	Mutant cell lines	KO; small indels	Indel mutant lines
Zuris et al., 2014	*EMX1; Tau-EGFP*	Neuronal marker	HEK293T; mESCs	Cationic lipid transfection of Cas9:sgRNA protein:RNA complex	Mutant cell lines	KO; small indels	Indel mutations; Loss of GFP signal
	*Atoh1-GFP; EMX1*	Hair cell marker in cochlea	Mouse, adult	Cochlear injection of cationic lipid prep of Cas9:sgRNA protein:RNA complex	Somatic and local alteration of genomic sequence	KO; small indels	Loss of GFP signal; indel mutations
Swiech et al., 2014	*Mecp2*	Rett syndrome	Mouse primary cortical neurons (E16)	AAV-Cas9 and AAV-sgRNA vector co-transduction	Mutant primary cells	KO; small indels	Indel mutant cells
			Mouse, adult	Hipp DG injection of AAV-Cas9 and AAV-sgRNA vectors	Somatic and local alteration of genomic sequence	KO; small indels	Indel mutations
	*Dnmt1, Dnmt3a, Dnmt3b*	Synaptic plasticity	Mouse, adult	Hipp DG injection of AAV-Cas9 and AAV-sgRNA vectors; multiplexing	Somatic and local alteration of genomic sequence	KO; small indels	Indel mutations
Incontro et al., 2014	*Grin1, Gria2*	Synaptic plasticity and transmission	Hipp slice or dissociated culture of postnatal rat	Biolistic co-transfection of Cas9 and sgRNA plasmids or lentiviral vector co-transduction	Mutant primary cells	KO; small indels	Indel mutant cells
Straub et al., 2014	*Grin1*	Synaptic plasticity	Mouse	Intrauterine electroporation of Cas9, sgRNA, and GFP plasmids	Somatic and local alteration of genomic sequence,	–	Functional analysis of KO through electrophysiology
			hipp slice culture of postnatal rat	Biolistic transfection (rat) of Cas9, sgRNA, GFP plasmids	Mutant primary cells	–	Functional analysis of KO through electrophysiology
Ponomareva et al., 2014	*clstn-1*	Axon branching, endosomal trafficking, AD	Zebrafish	1-cell embryo injection of TALEN mRNA	Germline transmission	KO; small indels	*clstn1^**uw*7*^* mutant line
Liu H. et al., 2014	*MECP2*	Rett syndrome	Rhesus and cynomolgus monkeys	Zygote injection of TALEN plasmids	Germline transmission	KO; small indels; SNPs	F0 mosaic mutants obtained
Richard et al., 2014	Artificial trinucleotide repeats	Neurodegeneration	Yeast	TALEN plasmid transformation in yeast strains	Yeast cells with sequence alterations	KO; deletions	Trinucleotide contraction
Liu Z. et al., 2014	*MECP2*	Rett syndrome	Cynomolgus monkeys	Zygote injection of TALEN mRNA	Germline transmission	KO; small indels	A F0 mosaic mutant stillborn
Niu et al., 2014^***^	*PPARG*	MS, AD, PD, ALS, and glioma	Cynomolgus monkeys	Zygote injection of Cas9 mRNA and sgRNAs; multiplexing	Germline transmission	KO; small indels	F0 mosaic mutants obtained
Huang et al., 2013	Tandem repeats [(TG)_n_]	Fragile X syndrome, HD, Friedreich's ataxia	Zebrafish	1-cell embryo injection of TALEN mRNA	Somatic mutants; mosaic animals	KO; deletions	Length change in (TG)_*n*_
Ferguson et al., 2013	*Tlr4*	Ethanol-induced neuroinflammation and behavioral effects	Rat	Zygote injection of TALEN mRNA	Germline transmission	KO; indels	*Tlr4^*13*del**^ mutants*
Li et al., 2013	*Th, Rheb, Mc4r*	Dystonia, TSC, BN	Mouse, rat	Zygote injection of sgRNA and Cas9 plasmid or RNA	Germline transmission	KO; indels	Indel mutants
Schmid et al., 2013	*tardbp, tardbpl*	ALS, FTLD-TDP	Zebrafish	Zygote injection of ZFN mRNA	Germline transmission	KO; indels	tardbp^−/−^ or tardbpl^−/−^ mutants
Cong et al., 2013	*EMX1, TH, PVALB*	Neurodev, dystonia, SCA1	HEK293FT	Cas9 and sgRNA plasmids co-transfection	Mutant cell lines	KO; indels	Indel mutants
Hwang et al., 2013	*apoea, gria3a, th1, rgs4, slc6a3, drd3*	AD, ID, schizophrenia, OCPD	Zebrafish	1-cell embryo injection of Cas9 and sgRNA plasmids	Germline transmission	KO; indels	Indel mutants

*Programmable DNA-binding agents (PDBAs; ZF, TALE, and Cas systems) are applied to knock-out target genes*.

* *Loci: APOE, apolipoprotein E; clstn1, calsyntenin 1, zebrafish homolog of human CLSTN1; Dnmts, family of DNA methyltransferases; DRD3, dopamine receptor D3; EMX1, empty spiracle homeobox 1; gba1, glucocerebrosidase 1, zebrafish orthologue of human GBA1; GFP, green fluorescence protein reporter; Gpr52, G protein-coupled receptor 52, mouse homolog of human GPR52; Gria2, glutamate ionotropic receptor α-amino-3-hydroxy-5-methyl-4-isoxazolepropionic acid (AMPA) type subunit 2, rat homolog of human GRIA2; GRIA3, glutamate receptor ionotropic AMPA subunit 3; Grin1, glutamate ionotropic receptor N-Methyl D-Aspartate (NMDA) type subunit 1, rat homolog of human GRIN1; Mc4r, melanocortin receptor type 4, rat homolog of human MC4R; Mecp2, methyl-CpG binding protein 2 gene, mouse homolog of human MECP2; Prnp, prion protein gene, mouse homolog of human PRNP; PVALB, parvalbumin gene; RGS4, regulator of G protein signaling 4; Th, tyrosine hydroxylase, mouse homolog of human TH; Rheb, ras homolog enriched in brain, mouse homolog of human RHEB; tardbp, transactive response DNA binding protein 43 kDa (TDP-43), zebrafish homolog of TDP-43, tardbpl, TDP-43 like; Tlr4, toll-like receptor 4, rat homolog of human TLR4. There are about 40 more studies that targeted neuronal loci that we could not discuss in this review*.

** *Implication: AD, Alzheimer's disease; ALS, amyotrophic lateral sclerosis; BN, bulimia nervosa; FTD, frontotemporal dementia; FTLD-TDP, frontotempmoral lobar degeneration with TDP-43 inclusions; GD, Gaucher's disease; glioma, neoplasm in the central nervous system; HD, Huntington's disease; ID, intellectual disability; Neurodev, neurodevelopment; PD, Parkinson's disease; SCA1, spinocerebellar ataxia type 1; TSC, tuberose sclerosis*.

*** *Two other non-neuronal loci are also targeted along with PPARG in this study*.

*C2C12, mouse myoblast cell line; Cas, CRISPR-associated; DG, dentate gyrus; ESCs, embryonic stem cells; F0, PDBA-injected generation; HEK293, human embryonic kidney cell derivative line; hESCs, human ESCs; indels, insertion and deletion mutations; Hipp, hippocampus; HSV, herpes simplex virus; herpes simplex viral vector; iPSCs, induced pluripotent stem cells; KI, knock-in; KO, knock-out; mESCs, mouse embryonic stem cells; N2a, mouse Neuro 2a cell line derived from neuroblastoma; NMuMG, mouse mammary gland epithelial cell line; NSCs, neural stem cells; sgRNA, single guide RNA; TALENs, transcription activator-like effector nucleases; ZF, zinc finger; ZFN, zinc finger nuclease*.

**Table 2:** **Neuronal target loci: knock-in**.

**Refernces**	Locus^*^	Implication^**^	**System**	**Delivery**	**Goal**	**Perturbation**	**Results**
Wen et al., 2016	*DISC1*	Schizophrenia	iPSCs, forebrain neurons	TALEN and donor plasmid co-transfection in iPSCs	WT or mutant cell lines	KI; correction or introduction of patient mutation	*DISC1^*WT*^* or *DISC1^*4*bp*−*del**^*
Lenzi et al., 2015	*FUS*	ALS	iPSCs, spinal motoneurons	TALEN and donor plasmid co-transfection	Mutant cell lines	KI; codon replacement	*FUS^*P*525*L*^* mutant iPSCs and neurons
Vannocci et al., 2015	*FXN*	Friedreich's ataxia	HEK293	Donor and TALEN or Cas9/sgRNA plasmid co-transfection	Mutant cell lines	KI; Introduction of donor sequence	Inducible FXN mutants
Kiskinis et al., 2014	*SOD1*	ALS	iPSCs, spinal motoneurons	ZFN and donor plasmid co-transfection in iPSCs	Mutant cell lines	KI; correction of patient mutation	*SOD1^**A*4*V**^*→*SOD1^*WT*^*
Wainger et al., 2014	*SOD1*	ALS	Same method as Kiskinis et al., 2014	–	–	–	–
Chen et al., 2014	*SOD1*	ALS	iPSCs, spinal motoneurons	TALEN and donor plasmid co-transfection in iPSCs	Mutant cell lines	KI; correction of patient mutation	*SOD1^*D*90*A*^*→*SOD^*WT*^*
Sanders et al., 2014	*LRRK2*	PD	iPSCs, dopaminergic neurons	ZFN and donor co-transfection in iPSCs (method not disclosed, proprietary information)	WT cell line	KI; correction of patient mutation	*LRRK2^**G*2019*S**^ → LRRK2^**G*2019*S**^*
An et al., 2014	*HTT*	HD	HEK293, iPSCs	Cas9 WT, Cas9 nickase, or TALEN and donor plasmid co-transfection	Mutant cell lines	KI; introduction of mutation	*HTT^*97*Q**^*
Jones and Meisler, 2014	*Scn8a*	Epileptic encephalopathy	Mouse	Zygote injection of TALEN mRNA and donor plasmid	Germline transmission	KI; introduction of mutation	*Scn8a^**N*1768*D**^* mutant
Hruscha et al., 2013	*tardbp, tardbpl, C13H9orf72*	ALS, FTD	Zebrafish	1-cell embryo injection of Cas9 mRNA and sgRNA	Germline transmission	KO; small indels	Indel mutants
	*tardbp, C13H9orf72*	ALS, FTD	Zebrafish	1-cell embryo injection of Cas9 mRNA, sgRNA, and ssODNs	Germline transmission	KI; introduction of HA tag	HA tag insertion
Woodruff et al., 2013	*PSEN1*	AD	iPSCs, neurons	TALEN plasmid and ssODN co-transfection	Mutant cell lines	KI; introduction of patient mutation	*PSEN1^*Exon*9*skip*^*
Panda et al., 2013	*FUS*	ALS	Mouse	Zygote injection to pronuclei of TALEN mRNA and ssODNs	Germline transmission	KI; codon replacement	*FUS^**R*513*G**^* or *FUS^**P*517*L**^* mutant
	*3110043021Rik*	ALS (*C9orf72*)	Mouse	Zygote injection to pronuclei of TALEN mRNA	Germline transmission	KO; small indels	Indel mutants
Fong et al., 2013	*MAPT*	Tauopathy	iPSCs, dopaminergic, glutamatergic, or GABAergic neurons	ZFN and donor plasmid co-transfection in iPSCs	Mutant cell lines	KI; correction or introduction of patient mutation	*MAPT^**A*152*T*/*A*152*T**^* or *MAPT^*WT*/*WT*^*
Zu et al., 2013	*th*	Dystonia	Zebrafish	1-cell embryo injection of TALEN mRNA and donor plasmids	Germline transmission	KI; introduction of reporter (EGFP)	EGFP insertional mutants
Bedell et al., 2012	*crhr1*	HPA axis modulation, anxiety	Zebrafish	1-cell embryo injection of TALEN mRNA	Germline transmission	KO; small indels	Indel mutants
	*crhr2*	Eating disorder		1-cell embryo injection of TALEN mRNA and ssODN	Germline transmission	KI; introduction of LoxP sequence	Mutants
Soldner et al., 2011	*SNCA*	PD	hESCs, iPSCs	ZFN and donor plasmid co-transfection	Mutant or WT cell lines	KI; introduction or correction of patient mutation	*SNCA^**A*53*T**^, SNCA^**E*46*K**^* mutant, *SNCA^*WT*^*

*Programmable DNA-binding agents (PDBAs; ZF, TALE, and Cas systems) are applied to knock-in exogenous sequences at target loci*.

* *Loci: 3110043021Rik, mouse homolog of the human C9orf72; C9orf72, chromosome 9 open reading frame 72; C13H9orf72, zebrafish homolog of the human C9orf72; DISC1, disrupted in schizophrenia 1; FUS, fused in sarcoma/translocated in liposarcoma; FXN, frataxin; HTT, huntingtin; MAPT, microtubule-associated protein Tau; MECP2, methyl-CpG binding protein 2 gene; PPARG, peroxisome proliferator-activated receptor gamma; Scn8a, sodium channel, voltage-gated, type VIII, alpha subunit; SNCA, alpha-synuclein, non A4 component of amyloid precursor; SOD1, superoxide dismutase 1; tardbp, transactive response DNA binding protein 43 kDa (TDP-43), zebrafish homolog of TDP-43; tardbpl, TDP-43 like; th, tyrosine hydroxylase, zebrafish homolog of human TH*.

** *Implication: AD, Alzheimer's disease; ALS, amyotrophic lateral sclerosis; FTD, frontotemporal dementia; glioma, neoplasm in the central nervous system; HD, Huntington's disease; PD, Parkinson's disease*.

*Cas, CRISPR-associated; ESCs, embryonic stem cells; F0, PDBA-injected generation; hESCs, human ESCs; HSV, herpes simplex virus; herpes simplex viral vector; indels, insertion and deletion mutations; iPSCs, induced pluripotent stem cells; KI, knock-in; KO, knock-out; sgRNA, single guide RNA; ssODNs, single strand oligodeoxyribonucleotides; TALENs, transcription activator-like effector nucleases; ZF, zinc finger; ZFN, zinc finger nuclease*.

Moving beyond dividing cells and germline mutations in mutant animals, four groups directly applied Cas9 systems in post-mitotic neurons in which two synaptic proteins, an NMDA receptor subunit (Incontro et al., 2014; Straub et al., 2014) and an AMPA receptor subunit (Incontro et al., 2014), are targeted (Table 1). Straub et al. locally altered the genomic sequence in a *Grin1* locus (NMDA receptor subunit 1) *in vivo* in E15 mouse hippocampi by delivering Cas9 and sgRNA plasmids through in-utero electroporation, as well as in hippocampal slice culture through biolistic transfection (Straub et al., 2014). The resulting neurons showed altered electrophysiological profiles providing functional evidence that the target gene, *Grin1*, is knocked down. However, mutations were not directly sequenced. Incontro et al. knocked out either *Grin1* or *Gria2* (AMPA subunit 2) in hippocampal slice culture from 6 to 11-day-old rats by delivering Cas9 and sgRNA plasmids through biolistic transfection (14-day expression of the construct before electrophysiology; Incontro et al., 2014). Incontro et al. showed that electrophysiological profiles were altered in the knock-out cells. They also reported that >90% of the mutations in *Grin1* had indel mutations that were out-of-frame and that the function of the target protein was 100% altered in all cells in which Cas9 expression was confirmed (Incontro et al., 2014). The findings by Incontro et al. imply that NHEJ could be the dominant mode of DNA DSB repair in post-mitotic neurons and that mutagenesis in neural cells could be much higher compared to other somatic cells because their post-mitotic nature does not allow the dilution of PDBAs.

Similarly, Swiech et al. targeted *Mecp2* or DNA methyltransferases (*Dnmt1, Dnmt3a*, and *Dnmt3b*; via multiplexing) in post-mitotic neurons *in vitro* and *in vivo* by stereotactically delivering Cas9 and sgRNA in adeno-associated viral vectors to the hippocampal dentate gyrus in adult mice (Swiech et al., 2014; Table 1). In primary cortical neuron culture, ~70% of transduced cells showed knock-out of *Mecp2* by immunocytochemistry, western blot, and next generation sequencing. The *in vivo* knock-out efficiency was ~68% in purified nuclei of transduced cells (*in vivo* transduction efficiency of the vector was ~80%) from dissected brain tissue. The *Mecp2* mutations led to altered performance in a contextual fear-conditioning paradigm, reinforcing that the *in vivo* knockdown of *Mecp2* in hippocampal cells has neuronal and behavioral consequences. Swiech et al. also demonstrated that somatic *Mecp2* knockdown affects the functions of neural circuits by showing altered primary visual cortex (V1) function when *Mecp2* was targeted in the superficial layers of V1. Multiplexed targeting *in vivo* created mutations in *Dnmt3a, Dnmt1*, and *Dnmt3b* loci with ~75, ~75, and ~50% indel rates, respectively, when the three loci were targeted in a single transduction in the hippocampus (Incontro et al., 2014). Swiech et al. demonstrated that Cas9-mediated multiplexed gene targeting in the nervous system can be accomplished *in vitro, in vivo*, and in localized somatic application as well as through zygotic injections. Most recently, Zuris et al. demonstrated non-viral delivery of PDBAs in *in vitro* and *in vivo* post-mitotic neuronal cells (Zuris et al., 2014; Table 1). Zuris et al. delivered a Cas9:sgRNA protein:RNA complex in cationic lipid to the cochlear and altered the *EMX1* locus with ~20% efficiency in neuronal cells (the outer hair cell) in 1-day-old mice. These four cases represent diverse Cas9 systems delivered with DNA plasmids, viral vectors, or cationic lipids, which are directly applicable to post-mitotic neurons *in vitro* and *in vivo*. These work demonstrate the feasibility of neuronal genome engineering and provide evidence that the mutagenic efficiency is comparable to or higher than that in dividing cells.

In addition to neuronal applications of PDBA-mediated NHEJ, understanding and exploiting the properties of NHEJ are underway in many investigations. Beyond out-of-frame knock-outs, in-frame indel mutations also occur and may be useful to study the function of specific amino acid residues or putative protein domains (Sung et al., 2013). Microhomology-mediated end joining (MMEJ) often results in deletions or translocations (He et al., 2015). First shown in mammalian cells in 1986, MMEJ has become a useful tool to bias deletions toward frameshift mutations (Roth and Wilson, 1986). The property of MMEJ that allows researchers to better predict mutagenic outcomes might be particularly useful for neuroscientists since NHEJ–resulting in random mutations–appears to be more active compared to dividing cells. Several software options provide microhomology-based prediction functions to design TALENs and CRISPR/Cas9 gRNAs that are more likely to induce frameshift mutations (Neff et al., 2013; Bae et al., 2014a).

### HDR and HR: knock-ins

Unlike the NHEJ repair pathway, HR and some versions of HDR utilize an external DNA donor and repair the DNA damage without introducing erroneous sequences. A single-stranded oligo with short sequence homology (~50 nt) to the genomic DNA can act as a template for HDR, and a donor DNA construct with extended homology arms (hundreds to thousands of bps) can act as a template in HR. Applications of PDBA-mediated knock-ins in neuroscience have focused on engineering induced pluripotent stem cells (iPSCs) and generating animal models (Table 2). First, it has been a widely-used and effective approach to engineer patient-derived iPSCs with PDBAs to revert a disease-causing allele to wild-type via HDR and to differentiate these engineered and control (without modification) iPSCs to neurons. The genome engineered iPSCs and neurons are identical to the patients' cells except the corrected DNA sequences and a certain level of single nucleotide polymorphisms (SNPs) inherent to all stem cell models. Patient-derived iPSCs have been used to investigate altered cellular functions and establish cell-autonomous disease models in Parkinson's disease (PD), tauopathy, amyotrophic lateral sclerosis (ALS), Alzheimer's disease, and Huntington's disease (HD) using ZFN (Soldner et al., 2011; Fong et al., 2013; Kiskinis et al., 2014; Sanders et al., 2014), TALEN (Woodruff et al., 2013; Chen et al., 2014; Lenzi et al., 2015; Wen et al., 2016), and Cas9 systems (An et al., 2014; Table 2).

Whereas a knock-in approach had been challenging and time-consuming in animal models, PDBAs have spurred the generation of knock-in cell and animal models based on increased efficiencies of HR. When considering the rates of classic HR to be ~1 × 10^−3^ and 1 × 10^−6^ (Smithies et al., 1985; Thomas and Capecchi, 1986; Puchta et al., 1993; Rouet et al., 1994; Choulika et al., 1995; Rong and Golic, 2001), PDBAs can increase donor sequence integration at a rate of ~1–45% by generating DSBs in the genomic target locus (Urnov et al., 2005; Beumer et al., 2008; Greenwald et al., 2010; Li et al., 2013; Zu et al., 2013; Gratz et al., 2014; Krentz et al., 2014; Platt et al., 2014; Shin et al., 2014a; Xie F. et al., 2014a; Karakikes et al., 2015; Lee et al., 2015; Wang et al., 2016). Although direct comparison is nearly impossible because of the differences in the knock-in system, including the size and design of the donor, selection systems, and target loci in the genome, targeted DSBs with PDBAs are viewed as improving HR rates by 3–6 orders of magnitude. Bedell et al. first showed *in vivo* knock-in of modified LoxP sequences in a neuronal locus, *crhr2*, by zygote injection of TALEN mRNA and a single-stranded oligonucleotide in zebrafish (Bedell et al., 2012; Table 2). Currently, patient allelic variants of many neuronal disorders have been introduced and modeled in mice and zebrafish using TALEN (Panda et al., 2013; Jones and Meisler, 2014) and Cas9 systems (Hruscha et al., 2013) ranging from epilepsy to schizophrenia (Table 2).

Directly knocking-in exogenous DNA sequences with PDBAs in post-mitotic neurons has not been reported to our knowledge. It will be an important area of investigation when developing therapeutics, and the mechanism of foreign DNA knock-in is being pursued to improve future therapeutic applications. It is important to recognize that the NHEJ, HDR, or HR pathways all can be used to introduce exogenous DNA sequences into the genome. A linear piece of double-stranded DNA may be incorporated into the genome even during NHEJ repair (Lin and Waldman, 2001; Miller et al., 2004; Gabriel et al., 2011). This NHEJ process is still prone to errors at the break site and the mechanism is unclear. Inactivating key components of the NHEJ pathway (e.g., ligase IV) has been demonstrated to bias DSB repair away from NHEJ, in one example increasing HR efficiency by 20–65% in *Drosophila* (Beumer et al., 2008). Another way to circumvent the NHEJ pathway is by generating single-strand overhangs with nickases and providing a double-stranded oligo with complementary overhangs on either side (Ran et al., 2013). Albeit with lower efficiency, HR-based knock-ins with short or long homology arms allow many experimental manipulations—including the introduction of promoters, reporters, recombination sites, and wild-type alleles—with high accuracy and are utilized in neuroscience research (Liu et al., 2011; Corti et al., 2012).

## Epigenetic modification and transcriptional control

There are estimated to be hundreds to thousands of types of neuronal cell populations (Stevens, 1998; Shin et al., 2014b) in the mammalian cortex, suggesting equally complex epigenetics in the nervous system. Development, physiological function (such as memory consolidation), and pathological changes in neuropsychiatric and neurodegenerative disorders are closely tied to epigenetic changes (for a review, see Tsankova et al., 2007; Morris and Monteggia, 2014; Reul, 2014). In recent years, we have significantly broadened our understanding of the three-dimensional structures and organizations of chromatin in the nucleus (Takizawa and Meshorer, 2008; Lieberman-Aiden et al., 2009; Phillips-Cremins et al., 2013; Rao et al., 2014). Similar to how the deluge of genomic sequencing data has urged the functional study of disease-associated and candidate genes, we are in need of functional understanding of higher chromatin structures and their connection to neuronal diseases. The ability to perturb epigenetic states, gene expression patterns, and higher order chromatin structures in a targeted manner has been largely unavailable. Although it is still at a fledging state, we will discuss multiple investigations that have reported success in direct modulation of gene expression, epigenetic modulation, building synthetic circuits, and modifying regulatory elements in mammalian cells, yeasts, and prokaryotes using PDBAs. The majority of the studies were conducted in dividing cells, and only a small portion of the studies targeted neuronal loci. Still, these early studies demonstrate the feasibility of controlling regulatory elements with PDBAs and are worth a discussion for their future adoption in the nervous system.

### Regulation of gene expression

**Figure 2:**
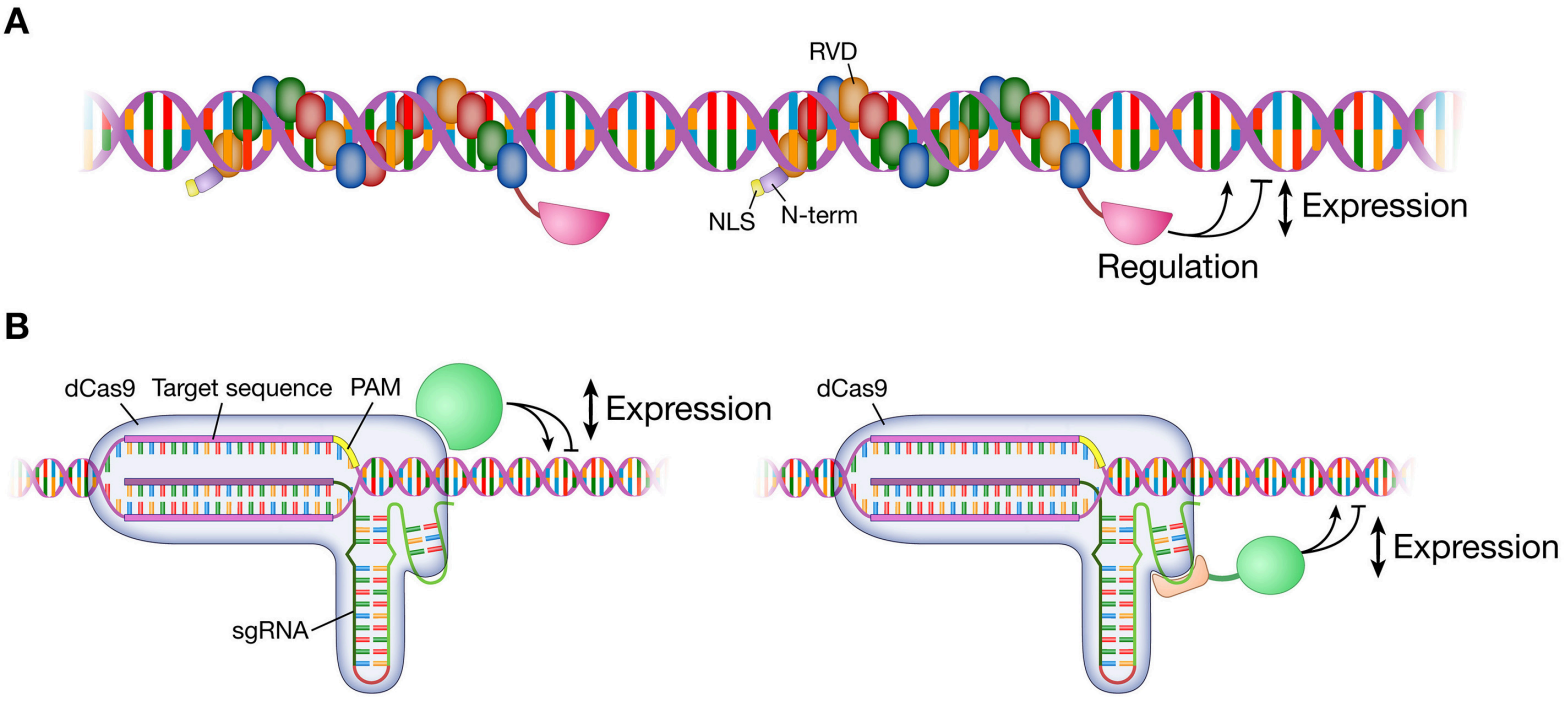
**Modulation of gene expression**. Targetable TALE and dCas9 fused to various effector domains modulate gene expression at the target locus in the genome. Effector domains can be transcription activator domains (e.g., VP64) or repressor domains (e.g., KRAB). A TALE monomer/sgRNA or multiple TALEs/sgRNAs are targeted to a locus for a range of gene expression modulation. **(A)** An effector domain is fused to the C-terminus of a TALE. **(B)** An effector domain is fused to or interacts with either dCas9 protein or sgRNA. TALE, transcription activator-like effector; dCas9, dead Cas9; catalytically inactivated Cas9 nuclease; sgRNA, single guide RNA; VP64, tetrameric repeat of herpes simplex virus activation domain (VP16); KRAB, Krüppel-associated box repression domain.

As the name implies, TALEs were first identified and adapted as transcription factors (Bai et al., 2000; Gu et al., 2005; Boch et al., 2009; Moscou and Bogdanove, 2009). Applying this inherent functionality, TALEs have been used to up-regulate the expression of specific genes at targeted loci (Zhang et al., 2011; Figure 2A). The application of single TALEs showed a moderate effect of two to five-fold increases in target gene expression in endogenous loci (SOX2, KLF4) in HEK293T cells, but the fold increase could be augmented from 50- to 10,000-fold by applying multiple TALEs targeting an endogenous promoter (Perez-Pinera et al., 2013b; Maeder et al., 2013c). Replacing the TALE's native activation domain with a stronger activation domain (VP16 and later VP64) increased activation by 10 times in a reporter system (Geiβler et al., 2011). In addition, TALEs have been shown to repress gene expression when fused to a repressor domain such as the EAR-repression domain (SRDX) in plants (Mahfouz et al., 2011) and the Krüppel-associated box (KRAB) or Mad interaction domain (SID) in human cells (Cong et al., 2012). Yet, the modulatory effects of TALEs are inconsistent. Some TALEs show a higher activity while some fail to modulate the transcription of the target locus. Although this ineffectiveness was attributed to repressed regions of chromatin (Zhang et al., 2011; Bultmann et al., 2012), other studies showed that TALEs can activate transcription even in repressed chromatin regions (Scott et al., 2014b). One explanation for this discrepancy is the location of the TALE binding site in relation to nucleosome binding. In particular, the activity of TALEs was higher when targeting DNA sequences that are accessible in the nucleosome, while not as efficient when targeted to sites covered by the dyad of the nucleosomes (Scott et al., 2014a). Three studies using TALENs reported modulation of gene expression in neuronal loci (Table 3). Cong et al. showed ~5- and three-fold up-regulation of mRNA in *CACNA1C* target loci 1 and 2 with TALE-VP64 in HEK293FT cells (Cong et al., 2012). Targeting *NTF3* in HEK293 cells, Miller et al. reported a ~30-fold increase in mRNA expression with TALE-VP16 (Miller et al., 2011) whereas Maeder et al. saw only a modest increase in the same gene with TALE-VP64 (though significant; Maeder et al., 2013c). These two cases show that, even in the same locus, the efficacy of TALE-mediated gene expression may be affected by multiple variables such as target loci, design and concentrations of TALE constructs, cell types, delivery methods, and the genetic make-up of transfected cells or injected animals. Similarly, different types of neurons in varying animal models could respond differently to the application of TALEs.

**Table 3:** **Neuronal target loci: gene expression**.

**References**	Locus^*^	Implication^**^	**System**	**Delivery**	**Goal**	**Perturbation**	**Results**
Amin et al., 2015	*miR-218-1*; miR-218-2^***^	Motoneuron disease	Mouse	Zygote injection of Cas9 mRNA and two sgRNAs	Germline transmission	KO; deletion	*miR-218-1^*370*bp*−*del**^* or *miR-218-2^*290*bp*−*del**^* mutant
Zuris et al., 2014	*NTF3*	Neurodegeneration	HEK293T	Cationic lipid transfection of (-30)GFP-TALE-VP64 protein or dCas9-VP64:sgRNA protein:RNA complex	Transient alteration of gene expression	–	Increased gene expression
Heller et al., 2014	*Fosb*	Addiction, depression	Mouse (juvenile 7-8 wks old)	HSV transduction of ZF+effector (p65 or G9a) plasmids	Transient and local alteration of gene expression	Acetylation or methylation of promoter	Up or down-regulation of gene expression
				HSV transduction of TALE+effector (VP64) plasmids	Transient and local alteration of gene expression	–	Increased gene expression
Konermann et al., 2013	*Neurog2, Grm2+27 other neuronal genes*	Neural differentiation, PD, Schizophrenia	N2a, mouse primary cortical neurons, mouse	Transfection of light-inducible TALE plasmids; transduction of light inducible AAV-TALE vectors	Light-induced transient alteration of gene expression	–	Increased gene expression with an improved temporal control
Maeder et al., 2013b	*NTF3*	Neurodegeneration	HEK293	dCas9-VP64 and sgRNA plasmid co-transfection	Transient alteration of gene expression	–	Increased gene expression
Chapdelaine et al., 2013	*FXN*	Friedreich's ataxia	Human and mouse fibroblast	TALE-VP64 plasmid transfection	Transient alteration of gene expression	–	Increased gene expression
Maeder et al., 2013c	*NTF3*	Neurodegeneration	HEK293	TALE-VP64 or TALE-p65 plasmid transfection	Transient alteration of gene expression	–	Increased gene expression
Garriga-Canut et al., 2012	*HTT*	HD	ST*Hdh* cells, mouse	ZFP plasmid or AAV2/1 vector transfection;	Transient or stable alteration of gene expression	–	Decreased gene expression
Cong et al., 2012	*CACNA1C*	Autism	HEK293FT	TALE-VP64 plasmid transfection	Transient alteration of gene expression	–	Increased gene expression
Miller et al., 2011	*NTF3*	Neurodegeneration	HEK293	TALE-VP64 plasmid transfection	Transient alteration of gene expression	–	Increased gene expression
Laganiere et al., 2010	*GDNF*	PD	Human, monkey, and rat cells	Lentiviral transduction of ZF-p65	Alteration of gene expression	–	Increased gene expression
			Rat	AAV2 transduction of ZF-p65		–	

*Programmable DNA-binding agents (PDBAs; ZF, TALE, and RAN-guided Cas systems) are applied to modulate target gene expression*.

* *Loci: CACNA1C, calcium channel voltage-dependent L-type alpha 1C subunit; Gdnf, glial cell line-derived neurotrophic factor, rat homolog of human GDNF; Grm2, glutamate receptor metabotropic 2, mouse homolog of human GRM2; HTT, huntingtin; miR-218-1, miR-218-2, microRNA-218 mammalian paralog 1, 2; Neurog2, neurogenin 2, mouse homolog of human NEUROG2; NTF3, neurotrophin 3*.

** *Implication: HD, Huntington's disease; PD, Parkinson's disease*.

*** *miR-218 and miR-218-2 are knocked out through deletion (370 bp, 290 bp). This study by Amin et al. is included in gene expression modulation section because the indels in the regulatory elements mainly concern gene expression patterns*.

*(-30)GFP-TALE-VP64, engineered GFP protein with electrostatic charge of -30 fused to TALE-VP64; AAV2, adeno-associated virus type 2; Cas, CRISPR-associated; dCas, dead Cas; enzymatically inactive; G9a, histone methyltransferase, encoded by EHMT2, euchromatic histone-lysine N-methyltransferase 2; HA tag, human influenza hemagglutinin amino acids 98–106 epitope tag; HEK293, human embryonic kidney cell derivative line; HSV, herpes simplex virus; herpes simplex viral vector; indels, insertion and deletion mutations; KO, knock-out; N2a, mouse Neuro 2a cell line derived from neuroblastoma; p65, NF-κB activation domain; sgRNA, single guide RNA; STHdh, neuronal progenitor cell line from mouse embryonic day 14 striatal primordial, first exon of the mouse Htt gene has been replaced by a human HTT exon with 111 CAG repeats; TALE, transcription activator-like effector; TALENs, transcription activator-like effector nucleases; VP64, tetrameric repeat of herpes simplex virus activation domain (VP16); ZF, zinc finger; ZFN, zinc finger nuclease*.

Although it is useful to be able to modulate gene expression levels at target loci by simply introducing TALEs to the system, these applications act constitutively and lack spatiotemporal control. Inducible TALEs and TALEs with regulated expression systems were developed to provide further spatiotemporal control. Allowing for increased temporal regulation, TALEs bound to a ligand-binding domain could modulate gene expression in response to light (Konermann et al., 2013), endogenous signals (Li et al., 2012), or exogenous ligands (Mercer et al., 2014). The study by Konermann et al. is noteworthy since the authors not only modulated two neuronal targets in an inducible system in dividing cells, primary neuron culture, and *in vivo*, but also profiled a total of 28 TALE-VP64 systems in primary neuron culture, each of which individually targeted one locus (Konermann et al., 2013). Konermann et al. built a two-hybrid system that allows TALE-mediated gene activation only when blue light (466 nm) is applied. One component of the two-hybrid is a TALE monomer bound to cryptochrome 2 protein (CRY2) effector domain that is sensitive to light and the other comprises a CRY2's interacting partner (C1B1 protein), nuclear localization signal, and VP64. With light stimulation, *Neurog2* mRNA is significantly increased from 30 min onward until about 3 h after stopping the stimulation in N2a cells (mouse neuroblastoma cell line), reaching a peak induction of 20-fold compared to that of unstimulated cells. After AAV-mediated delivery of inducible constructs targeting a *Grm2* locus in mouse primary cortical neurons and mice, Konermann et al. observed ~four- to six-fold increase in *Grm2* mRNA after light stimulation in neuronal culture and ~3.5-fold increase *in vivo* (Table 3). In a light-inducible repressor system, Konermann et al. fused CRY2 with mSin3 interacting domains that repress gene expression through histone deacetylation and observed two-fold reduction in *Grm2* mRNA expression in primary neuron culture (Konermann et al., 2013). Non-inducible TALE-VP64 constructs were tested in neurons as well by individually targeting 28 neuronal loci with a significant fold increase ranging from ~1.5 (*Grin2a*) to ~33 (*Slc6a4*).

To generate controlled and coordinated gene circuits, TALEs that interact with regulatory elements were developed, including TALEs that control synthetic promoters or TALEs that are expressed under the control of an artificial regulatory element. Multiple synthetic promoters that contain a TALE-binding site simultaneously activated gene expression in multiple loci in plants (Brückner et al., 2015), which showed the potential for a coordinated control of multiple genes. A similar system was developed to allow for a temporal regulation of TALE expression and circuit-level control of multiple genes in prokaryotes (Rai et al., 2015). RiboTALEs incorporate riboswitches upstream of the TALE-encoding sequences to regulate the time at which TALEs are expressed. Once expressed, TALEs repress gene expression of target loci that are engineered to have a TALE-binding site (Rai et al., 2015). Improved spatiotemporal control was also demonstrated in mammalian cells by combining the well-established tetracycline-response element (TRE) and Gal4-UAS system with TALEs fused to a repressor domain (Li Y. et al., 2015). Combining a TALE-repressor with other regulatory systems—in this case, TRE and Gal4-UAS—produced a synthetic circuit via a two-hybrid regulatory system.

Similar modulations of gene expression were achieved by engineered CRISPR/Cas systems. Unlike TALEs, the innate function of the CRISPR/Cas system is to make DNA DSBs as a bacterial adaptive immune system in nature (Barrangou et al., 2007; Garneau et al., 2010). To co-opt the CRISPR/Cas system as a transcription factor system, the innate endonuclease function of Cas9 is catalytically inactivated (dCas9; dead Cas9) by introducing D10A and H841A mutations within the nuclease domains, RuvC1 and HNH, respectively (Jinek et al., 2012; Figure 2B). The resulting dCas9 without any fused effector domains tends to block transcription in endogenous loci in prokaryotes and a reporter system in human cells, presumably through steric hindrance to the binding or elongation of the RNA polymerase (RNAP; Qi et al., 2013). dCas9 fused to a repression domain (KRAB) effectively silenced gene expression both in endogenous loci (CD71 and CXCR4) and in a reporter system. When fused to an activating domain (VP64), dCas9 increased GFP expressions in a reporter system (Gilbert et al., 2013). It was not reported if there was an activation in an endogenous locus (Gilbert et al., 2013). A neuronal endogenous locus was targeted with a dCas9-VP64 system showing a moderate (significant) level of increase in *NTF3* mRNA (~three-fold) at a concentration of 200 ng sgRNA and 250 ng dCAs9-VP64 and a high increase (~5–25) at a concentration of 500 ng sgRNA and 500 ng dCas9-VP64 (Maeder et al., 2013b). This study indicates that applying an optimal concentration without toxicity is an important factor. Robust activation of endogenous gene expression (ranging from 8- to 2000-fold) in human cells (Cheng et al., 2013; Maeder et al., 2013b; Mali et al., 2013a; Perez-Pinera et al., 2013a) and in mouse zygotes (Cheng et al., 2013) was later reported, yet a combination of multiple sgRNAs for each locus was necessary to see effective activation of the target gene in all studies. Mali et al. engineered sgRNA to include MS2 bacteriophage coat protein-binding RNA stem loop at the 3′ end and function as a effector carrying molecule (Mali et al., 2013a). This approach still required multiple sgRNAs to significantly activate an endogenous gene. This is consistent with reports that multiple TALEs function better at increasing the transcription of target locus from two- to five-fold of a single TALE to 50- to 10,000-fold in multiple TALEs.

The inability of the CRISPR/dCas9 system to activate gene expression with one sgRNA was recently amended with an alternative approach. Instead of linking effector domains to dCas9, two groups independently engineered the sgRNA secondary structure allowing the binding of effector domains to an sgRNA motif similar to the approach by Mali et al. (Figure 2B). Zalatan et al. incorporated viral RNA sequences of MS2, PP7, and com into sgRNA (this RNA guide is referred to as scaffold RNA; scRNA) that are recognized by the MCP, PCP, and Com RNA-binding proteins, respectively (Zalatan et al., 2015). The MCP, PCP, and Com proteins are fused to an activation (VP64) or repression (KRAB) domain and operate as an RNA-binding adaptor for the effector. This system expanded the two-factor system of sgRNA and dCas9 to a three-factor system of scRNA, dCas9, and adaptor-effector. Utilizing this system, each scRNA activated or repressed a different target locus mediated by different combinations of the adaptor and effector complex, maximizing the orthogonal control of gene regulation (Zalatan et al., 2015). Konermann et al. engineered sgRNA to incorporate dimerized MS2-binding sequences, made an adaptor-effector complex comprising MS2-p65-HSF1, and kept the fused VP64 activation domain on dCas9 (this system is currently referred to as a synergistic activation mediator; SAM; Konermann et al., 2015). Each group showed that one gRNA-mediated targeting could increase gene expression in endogenous loci from ~5-fold (Zalatan et al.) to ~15-fold (Konerman et al.). Zalatan et al. demonstrated orthogonal regulation by simultaneously activating and silencing gene expression, and Konerman et al. introduced an optimized system in activating gene expression.

Besides immortalized human cell lines, the CRISPR/Cas system was tested for gene activation and silencing in prokaryotes (Bikard et al., 2013) and human pluripotent stem cells (hPSCs; Kearns et al., 2013). In *Escherichia coli*, dCas9, fused to an RNAP omega subunit that stabilizes RNAP at the proximal promoter, increased β-galactosidase activity in a reporter system by ~three-fold (Bikard et al., 2013). In hPSCs, one sgRNA increased endogenous gene expression (SOX17) by 287-fold (Kearns et al., 2013), which was the highest fold increase with one sgRNA in CRISPR/dCas9 applications reported to date in any human cell line or prokaryote. This finding might implicate that distinct regulatory landscapes and mechanisms in different cell lines determine the performance of dCas9 and that CRISPR/dCas9 may be useful to differentiate hPSCs to various types of somatic cells.

Still, when considering effective gene regulation, maximizing the inherent function of each reagent appears to be the best route. In all studies that we have reviewed as of 2015, excepting the report by Kearns et al. TALEs showed a better efficiency in activating endogenous gene expression than did CRISPR/dCas9 in both one and multiple TALE-monomer/sgRNA applications. Gao et al. investigated the efficiency of gene activation between a TALE construct and four different constructs of dCas9 which targeted enhancer regions in Oct4 and Nanog loci (Gao et al., 2014). The report showed that a TALE monomer activated either reporter or endogenous genes significantly better than did any construct of dCas9 with one sgRNA (Gao et al., 2014). Importantly, Gao et al. increased the rigor of the experiment from reporter expression to endogenous gene expression and to cellular reprograming differentiating mouse embryonic fibroblasts (MEFs) to iPSCs. dCas9s could not produce any reprogrammed cell colonies even though they could increase the reporter and endogenous gene expression, whereas TALEs produced reprogrammed iPSC colonies (Gao et al., 2014). On the other hand, dCas9 constructs had equivalent or better activity in silencing gene expression than did a TALE monomer (Gao et al., 2014). Gao et al. speculate that the innate steric hindrance that dCas9 shows at the target locus might interfere with the endogenous transcription factor machinery, limiting the capacity of dCas9 to activate gene expression.

Such strengths of dCas9 in silencing gene expression had been shown in an early study in which dCas9 without a repressor domain could override the effect of doxycycline-inducible rtTA (tetracycline transactivator) targeting the same tet operator sequence as the sgRNA (Gilbert et al., 2013). The potent gene repression by dCas9 has been applied to modify the lac regulatory pathway in *E. coli* (Qi et al., 2013), to reprogram complex biological pathways in yeasts (Zalatan et al., 2015), and to build two-hybrid regulatory circuits with the Gal4 system in mammalian cells (Kiani et al., 2014).

### Modification of regulatory elements and epigenetic environment

In addition to the direct regulation of transcription, one can target and modify epigenetic environments and chromatin organizations to investigate important mechanisms of neuronal function. For example, TALEs fused to LSD1 histone demethylase demethylated histones at endogenous enhancer loci and subsequently down-regulated expression of the genes under the control of the targeted enhancer in human cell lines (Mendenhall et al., 2013). In another example, investigations from the Gersbach group using dCas9 fused to an effector domain showed robust modification of histone epigenetic markers. Hilton et al. fused dCas9 to the catalytic domain of human acetyltransferase p300 and observed robust up-regulation of H3K27 acetylation (four- to eight-fold) and of target gene expression (5- to 265-fold; Hilton et al., 2015). Thakore et al. investigated effects of KRAB repressor in epigenetic landscape and found enriched H3K9 trimethylation specifically at the target locus (HS2 enhancer) and consequent gene silencing of multiple globin genes under control of HS2 (Thakore et al., 2015). In addition to histone modifications, the methylation status of DNA contributes to the epigenetic environment. TET1 hydroxylase is responsible for the first step of 5-methylcytosine demethylation, which often activates a promoter (Shin et al., 2014b). This, too, can be modified with PDBAs, and targeted demethylation of CpGs in the promoter by TALEs fused to a TET1 hydroxylase catalytic domain increased the expression in endogenous target loci in human cells (Maeder et al., 2013a).

The genome has higher organizational structures beyond localized epigenetic environments. The multigene complex is one of the higher organizations and represents the locus on the genome where multiple genes on the same or different chromosomes associate with RNA polymerase (RNAP). The transcription of these multiple genes can be synchronously controlled at this multigene complex. Introducing mutations to a multigene complex using TALENs disrupted gene loop formation and significantly decreased gene expression (Fanucchi et al., 2013). Targeting Cas9 with two sgRNAs to the regulatory sequences, Li et al. demonstrated that efficient inversions and duplications of the regulatory elements and gene clusters can be induced in human cells and mice by inducing 2–4 DNA DSBs separated by a few tens of bases to several hundred kilo bases (Li J. et al., 2015). This perturbation was transmitted to offspring and led to the discovery of a new regulatory role of the protocadherin gamma gene cluster.

## Visualization

Besides specific manipulation of genomic sequences, PDBAs can be adapted to visualize the native organization of the chromatin. Alterations in DNA structures are implicated in multiple trinucleotide repeat disorders such as Huntington's disease and spinocerebellar ataxia (Kovtun et al., 2001) as well as hexanucleotide (GGGGCC) repeats in amyotrophic lateral sclerosis (ALS) and frontotemporal dementia (FTD; Haeusler et al., 2014). Using new PDBA reagents, genome organization in these disorders may be visualized in live cells. Using TALEs fused to fluorescent proteins (TALE-FP), Ma et al. imaged repetitive DNA sequences in the telomere of live or fixed human cells (U2OS cells; Ma, H. et al., 2013b). A specific chromosome was identified using centromeric repeat sequences specific to the particular chromosome (Ma, H. et al., 2013b). Miyanari et al. used TALE-FP to show that paternal and maternal chromosomes could be differentially labeled and visualized in live mouse cells and embryos using single nucleotide polymorphisms (SNPs) in the chromosomes due to the extreme sequence specificity of TALEs (Miyanari et al., 2013). Using a dCas9 system fused to fluorescent proteins and capitalizing on the strength of the CRISPR/dCas9 system for multiplexing, Chen et al. visualized non-repetitive genomic sequences by “tiling” sgRNA along the target locus (Chen et al., 2013). All three studies imaged changing chromosomal organizations at each cell cycle. It is not known whether and how post-mitotic neurons change genomic conformation. The three studies indicate that the structural and organizational understanding of the genome in neurons could be done *in vivo*.

## Off-target effects

In each application of PDBAs, a strategy to identify off-target effects needs to be in place. Despite the precision that the PDBA systems have shown, it is imprudent to expect any PDBA to bind to one unique locus in the genome. Plant pathogenic bacteria in the genus *Xanthomonas* evolved type III secretion system delivering TALEs into plant cells to induce expression of the host genes beneficial to their survival (Yang and White, 2004; Gu et al., 2005). Several off-targets in DNA binding would not have compromised the purpose of the system. When bacteria detect foreign viral DNA sequences registered in their CRISPR sequences (Ishino et al., 1987; Barrangou et al., 2007; Garneau et al., 2010), a few mismatches might be tolerated as far as the exogenous DNAs are effectively cleaved in the same way the restriction endonuclease system has evolved in bacteria. Thus, the specificity of targeting a locus in the genome is a primary concern that arises with the adaptation of the biological systems to human needs, in particular, for therapeutic purposes. In most research systems, off-target effects of PDBAs can be ruled out by generating two independent mutant alleles in which a genotype-phenotype relationship is confirmed. In animal models, potentially undetected off-target effects can be diluted out through chromosomal shuffling during meiosis. In cell culture models, two clones with the same mutation can be chosen to ensure the phenotype appears due to the mutation. With therapeutic and clinical applications, the system needs to be highly specific. If a PDBA system is used somatically to alter DNA sequences in differentiated adult cells, the change is permanent in the individual. In germ-line applications, the changes to the genetic material are permanent and heritable. These serious implications in clinical applications need to be considered, but it is equally important to see that there are different levels of stringency for precision depending on the nature of the experiments planned. Therefore, for researchers who plan to use a PDBA system, their experimental purposes are the most important consideration when choosing a PDBA system. For genome engineering as a discipline, researchers have most intensely investigated two areas as they aim to develop therapies with PDBAs: one to improve the specificity of the system and the other to detect off-target sites and effects.

Statistically, a CRISPR/Cas or TALE (monomer with 15-RVDs) system can incur off-target effects (cleavage or altered expression), although a TALEN (15-RVDs × 2) system may offer unique binding even in large mammalian genomes including the human's. An sgRNA in the CRISPR/Cas system can encounter a binding sequence by chance every 4^12^ bases (~1.5 × 10^7^) to 4^14^ bases (~2.5 × 10^8^), taking into account two specific nucleotides in the PAM sequence (nGG) and 10–12 nt of seed sequences immediately upstream of the PAM sequence within a 20-nt sgRNA. This results in 11–179 potential binding sites in a human haploid genomic sequence (~3 gigabases) assuming random genomic sequences. Mismatches are tolerated even in the seed sequences in the sgRNA, which increases the chance of off-target binding of Cas9 further. A 15-RVD based and dimerized TALEN system would encounter a binding site every 4^30^ bases (~1.0 × 10^18^), which suggests the possibility of a unique target site in the human haploid genome. However, since the binding of each RVD is not completely exclusive to one nucleotide, TALENs also have detectable off-target effects (Hockemeyer et al., 2011; Fine et al., 2014).

Statistical probability notwithstanding, experimentally-determined data on off-target effects of the CRISPR/Cas system have been different from that of bioinformatics predictions. Methods to identify off-target effects have evolved from sequence-based computational prediction to genome-wide identification of Cas9-binding sites with chromatin immunoprecipitation (ChIP), and later to genome-wide interrogation of DNA cleavage (for a review, see Ishida et al., 2015; Koo et al., 2015; O'Geen and Yu, 2015). Sequenced genomes allow direct comparison of the nucleotide sequences of designed sgRNAs, identifying predictable off-target binding sites. This bioinformatic identification of off-target sites was the basis of early studies in which these selected sites were monitored with reporter systems and sequenced (Cradick et al., 2013; Hsu et al., 2013; Pattanayak et al., 2013; Mali et al., 2013a; Cho et al., 2014; Fu et al., 2014). The off-target binding and DNA cleavage resulting from these studies varied widely—depending on the target site and sgRNAs—from no off-target sites to ~10 sites for each sgRNA. It is not clear if this is a site-specific epigenetic environmental effect, sgRNA sequence effect, or something else. After this selected investigation of off-target effects, more unbiased approaches were developed. These investigations utilized RNA sequencing (Gilbert et al., 2013; Perez-Pinera et al., 2013a) or ChIP followed by sequencing (Cencic et al., 2014; Kuscu et al., 2014; Wu et al., 2014; Polstein et al., 2015). To investigate the binding of Cas9 protein to the target locus, a modified version of dCas9 was used with ChIP-seq applications. It was hypothesized that since dCas9 does not cleave the DNA and stays bound, it would generate unbiased genome-wide snapshots unmasked by potentially varying efficiency of DNA repair in different cell lines. The majority of the studies found a highly enriched binding to the target sites and highly specific gene expression patterns (0 or 1 off-target binding sites), yet there was identifiable off-target binding (up to a few hundred sites) for some sgRNAs as well. These methods presented two challenges. First, it was difficult to functionally determine if the binding of dCas9 changed gene expression since sequence mutations were absent. A threshold has to be arbitrarily set to interpret if the changes in gene expression were significant. Second, this method also captured transient binding of dCas9 to the genomic DNA. The mechanism of Cas9-DNA binding suggests that Cas9 scans the genome for the PAM sequence (5′-NGG-3′) and that there is transient binding depending on the degree of sequence matching between the sgRNA and genomic DNA sequence (Anders et al., 2014). Thus, the off-target binding of dCas9 might not be off-target events but merely the locus of dCas9 at the moment of the assay.

Overcoming these limitations, unbiased DNA cleavage event-based methods were developed. These methods integrate DNA double-strand break (DSB) events with insertion of reporters or capturing of the broken fragments with adaptors. IDLV (integrase-defective lentiviral vector), GUIDE (genome-wide, unbiased identification of DSBs enabled by sequencing), and BLESS (direct in situ breaks labeling, enrichment on streptavidin and next-generation sequencing) methods make use of lentivirus integration, double-strand oligo deoxyribonucleotide (dsODN), or streptavidin as a DSB capture device, respectively (Tsai et al., 2014b; Ran et al., 2015; Wang et al., 2015). While the strength of these methods is that DNA DSBs are captured, their shortcoming is that it might capture DSB events that are independent of PDBA-induced breaks. More recently, two technologies provided an improved way of identifying PDBA activities. O'Geen et al. co-opted a ChIP-seq based method to enrich significant dCas9-binding signal by designing capture probes (O'Geen et al., 2015). Kim et al. developed a system to identify off-targets without any capture devices by re-digesting Cas9-treated genomic DNA with Cas9 *in vitro* (Kim et al., 2015). These unbiased approaches find that CRISPR/Cas systems are specific, making one off-target cut in the whole genome when appropriately designed sgRNAs were used (O'Geen et al., 2015). For an sgRNA to be deemed appropriately designed, the sgRNA has to be unique in the genome by more than four nucleotides compared to potential off-target sites. However, there are cases of sgRNAs that led to several tens off-target sites despite the apparent soundness of the design (Tsai et al., 2014b; Kim et al., 2015; Ran et al., 2015; Wang et al., 2015). In addition, these methods confirmed that computational sequence predictions are different from actual off-target cleavage sites and that dCas9-binding sites are different from actual off-target cleavage sites. Some reasons for the differences identified are sequence variation in the sample (Yang et al., 2014) and DNA or sgRNA bulging at the target sites (Lin et al., 2014). These studies demonstrate that while CRISPR/Cas systems have clear off-targeting effects, they rarely have the spurious off-target sites that bioinformatic methods predicted. However, further optimization is absolutely required to adapt CRISPR/Cas systems for therapeutic purposes, as zero off-target sites is the expectation for clinical applications.

Efforts to increase the specificity of CRISPR/Cas9 systems have attempted various strategies. Optimized sgRNA designs such as shorter sgRNAs (17–18 nts) truncated at the 5′-end of conventional 20 nt sgRNAs (Fu et al., 2014) or addition of two guanine nucleotides at the 5′-end of sgRNAs (Cho et al., 2014) significantly decreased off-target effects. Cas9 has been engineered to make Cas9 variants with either endonuclease domain, RuvC or HNH, inactivated (Jinek et al., 2012). A pair of Cas9 nickases targeted to adjacent sites on opposite strands results in DNA DSBs with significantly reduced off-target effects (Ran et al., 2013; Mali et al., 2013a; Cho et al., 2014; Frock et al., 2014; Shen et al., 2014). In a similar pairing scheme, a dCas9 protein with nuclease domains inactivated is fused to FokI endonuclease, as FokI dimerization is required to make DNA DSBs. The dCas9-FokI systems showed significantly improved specificity (Tsai et al., 2014a; Guilinger et al., 2014b; Wyvekens et al., 2015). A disadvantage of nickase and dCas9-FokI systems is that the pairing requirement decreases the number of available target sites in a genome since two closely located PAM sequences are needed.

Most recently, two groups independently reported engineered *S. pyogenes* Cas9 (SpCas9) systems that show significantly decreased off-target effects using current unbiased genome-wide off-target screening technologies. Both groups optimized the energetics during the formation of target DNA-guide RNA-SpCas9 complex by decreasing non-specific interactions between SpCas9 protein and target DNA while maintaining specific interactions between guide RNA and the complementary strand of target DNA (Kleinstiver et al., 2016; Slaymaker et al., 2016). Slaymaker et al. focused on the non-specific electrostatic interactions between the negatively charged phosphate backbone of the non-complementary strand of target DNA and the positively charged amino acid residues in SpCas9 protein in the groove between two nuclease domains (Slaymaker et al., 2016). Three engineered SpCas9 variants [SpCas9 (K855A), eSpCas9(1.0) (K810A/K1003A/R1060A), and eSpCas9(1.1) (K848A/K1003A/R1060A)] showed undetectable off-target effects at three previously identified off-target sites using next generation sequencing in human cell cultures (Slaymaker et al., 2016). When all known off-target sites were investigated, eSpCas9(1.0) and eSpCas9(1.1) showed a significantly decreased cleavage rate (<0.2% indel) in 22 out of 24 sites. In BLESS assessments for unbiased genome-wide off-target screening, there were still noticeable indels while reduction in off-target cleavage was clear compared to wild-type Cas9. BLESS only detected 1.0 and 0.7% indels at two *EMX1*(1) off-target sites (OTs) and 2.5, 6.3, 14.7, and 25.5% at four *VEGFA*(1) OTs for SpCas9 (K855A). eSpCas9(1.1) showed no discernable indels at *EMX1*(1) and 0.3 and 27.0% indels at two *VEGFA*(1) OTs. The three SpCas9 variants [SpCas9 (K855A), eSpCas9(1.0), and eSpCas9(1.1)] maintained on-target indel efficiency comparable (30–160%) to wild-type SpCas9 assessed with 24 sgRNAs in 10 endogenous loci (Slaymaker et al., 2016).

While Slaymaker et al. focused on non-complementary DNA strand and electrostatic interactions, Kleinstiver et al. engineered the Cas9 amino acid residues that make non-specific contact with complementary DNA strand to sgRNA through hydrogen bonds (Kleinstiver et al., 2016). Kleinstiver et al. abrogated hydrogen bonding between the Cas9 residues (N497, R661, Q695, and Q926) and the phosphate backbone of the complementary strand of target DNA by replacing those residues with alanine residues. When assessed with GUIDE-seq for unbiased genome-wide off-target detection, the Cas9 variant, SpCas9-HF1, with all four residues replaced with alanine, showed a complete absence of off-target effects with seven sgRNAs in four endogenous loci in human cells and only one detectable off-target effect. Subsequent next generation sequencing of 36 wild-type Cas9 OTs identified in GUIDE-seq showed that SpCas9-HF1 had only a background level of indels at 34 out of 36 OTs and negligible indel rates of 0.049 and 0.037% at the other two OTs (Kleinstiver et al., 2016). SpCas9-HF1 maintained on-target mutagenic efficiency comparable (70–140% excluding one sgRNA with no activity) to wild-type Cas9 with 12 sgRNAs in five endogenous loci (Kleinstiver et al., 2016).

The differences in off-target effects between the two structure-guided protein engineering studies might come from varying sensitivity of genome-wide screening technologies (BLESS vs. GUIDE-seq). Importantly, these two studies demonstrated that rational protein engineering can improve specificity in CRISPR/Cas systems. Along with off-target detecting techniques and engineered Cas9 proteins, sgRNA designing software is evolving, which may further decrease off-target effects through design of higher-specificity sgRNAs (Bae et al., 2014b; Montague et al., 2014; Xie S. et al., 2014b; Moreno-Mateos et al., 2015).

Although statistics and bioinformatics predict that TALENs are highly specific, TALENs also have off-target effects. With optimization of the system, the number of off-target sites have significantly decreased (Osborn et al., 2013; Ousterout et al., 2013; Guilinger et al., 2014a). A study showed that the 5′-side of TALEN-binding site (N-term side of TALENs) is more influential in determining TALEN specificity and found that for higher activity and specificity, the first two bases should not be adenine-adenine or cytosine-adenine (Meckler et al., 2013; Juillerat et al., 2014). The degree of individual RVD specificity was also studied. All classic RVDs (NI:A, HD:C, NN:G, and NG:T) do not bind exclusively to one base; rather, RVDs have degrees of discriminating power among the four bases rather than an RVD exclusively binds to a base. For example, HD conventionally binds to cytosine (C) and discriminates C from adenine, guanine, and thymine with scores of 0.06, 0.80, and 0.47, respectively (where 1.00 represents perfect discrimination; Juillerat et al., 2015). This limited specificity led to the development of non-conventional RVDs to improve the specificity of TALENs targeting the same locus. In a different approach using modified phage display and protein evolution systems, Hubbard et al. developed a directed protein evolution system in which the DNA binding domain of TALENs can be rapidly optimized for an improved specificity (Hubbard et al., 2015). It is now critical to apply unbiased genome-wide screening developed for CRISPR/Cas9 systems to TALEN off-target screening to validate that directed protein evolution and non-conventional RVDs do indeed decrease off-target effects.

Acknowledging that none of the PDBAs are absolutely specific is important because it will facilitate investigations to improve specificity and detect off-target effects. As a practical guideline, any investigation using PDBAs should use an alternative design (two target sites against a locus) to show the genotype-phenotype relationship beyond doubt. In addition, large-scale projects should be mandated to employ at least one unbiased off-target screening method to show that the PDBA used is reasonably specific without any spurious off-target effects. Lastly, if the system is an animal model, F2 or later generations rather than F1s should be used to ensure the dilution of non-specific mutagenesis. If enough cautions are used, PDBAs are already precise enough to accommodate most (if not all) of research needs. For therapeutic applications, any PDBA systems will need to be further optimized. However, if the absence of off-target effects is shown in cell culture systems and animal models, the particular PDBA design that targets a specific locus may be tested in human subjects.

## Conclusions and perspectives

New generations of programmable DNA-binding agents (PDBAs) such as TALE and CRISPR/Cas systems have rapidly materialized highly efficient genome engineering systems. PDBAs can now target nearly any locus in the genome, introduce or correct mutations, modulate gene expression, and change epigenetic environments with a controllable level of off-target effects, which provides unprecedented opportunities to understand neuronal and neuropsychiatric disorders. These PDBA technologies are being incrementally introduced in neuroscience, and there are only a small number of investigations that focus on the effects and outcomes of PDBA application in the nervous system. Considering distinctive mechanisms of DNA double-strand break repair and epigenetic regulation in neurons, we need more studies to complete our understanding of the functions and effects of applying PDBAs in the nervous system. To access the central nervous system, delivery mechanisms will need to be further engineered (Holkers et al., 2014; Ain et al., 2015; Chen and Gonçalves, 2015). To achieve cell-line specific inducible PDBA systems, neural populations and cell-line specific promoters need to be better characterized to avoid false outcomes (Forni et al., 2006; Galichet et al., 2010). In spite of these outstanding challenges, PDBAs will be a pivotal technology that will lead to new understanding and drive innovations in neuroscience.

## Author contributions

All authors listed, have made substantial, direct and intellectual contribution to the work, and approved it for publication.

## Funding

The National Institutes of Health (Grants: GM63904, DK84567), Mayo Graduate School, the Mayo Clinic Center for Individualized Medicine, Sanford Research, and the Mayo Foundation for Medical Education and Research provided funding and support for our work.

### Conflict of interest statement

The authors declare that the research was conducted in the absence of any commercial or financial relationships that could be construed as a potential conflict of interest.
